
JOURNAL INFORMATION
==============================
NLM Title Abbreviation: Am J Trop Med Hyg
Journal ID: Am J Trop Med Hyg
NLM Journal ID: 0370507
Journal ID: tpmd

The American Journal of Tropical Medicine and Hygiene

ISSN: 0002-9637
EISSN: 1476-1645
Publisher: The American Society of Tropical Medicine and Hygiene

ARTICLE INFORMATION
==============================
PMCID: PMC11720688
DOI: 10.4269/ajtmh.1121stoc
Article ID: tpmd1121stoc
Article version: 1
Subjects: Other

Contents

Electronic publication date: 2025 Jan 7
Print publication date: 2025 Jan
Volume: 112
Issue: 1 Suppl
Issue title: Surveillance Data for Decision-Making in Global Health: Enhancing Analysis, Integration, and Action
First page: ii
Last page: ii
Copyright: © 2025, The American Society of Tropical Medicine and Hygiene
Copyright year: 2025
License: This is an open-access article distributed under the terms of the Creative Commons Attribution (CC-BY) License, which permits unrestricted use, distribution, and reproduction in any medium, provided the original author and source are credited.
License URL: https://creativecommons.org/licenses/by/4.0/

Page count: 1

==============================

### Surveillance Data for Decision-Making in Global Health: Enhancing Analysis, Integration, and Action

**Guest Editors: Debra Prosnitz and Yazoumé Yé**

### CONTENTS

**EDITORIAL**

**Harnessing Malaria Surveillance Data for Transformative Malaria Control and Elimination**
Yazoumé Yé and Debra Prosnitz **1**

**ARTICLES**

**Identifying Strengths and Gaps in Data Management and Reporting through Malaria Routine Data Quality Assessment: Results from Two Health Regions in Madagascar**
Maurice Yé, Jean Marie N'Gbichi, Tokinirina Andrianantoandro, Sabas Rabesahala, Urbain Rabibizaka, Brune Estelle Ramiranirina, Omega Rabeola, Laurent Kapesa, and Yazoumé Yé **3**

**Evaluating the Implementation of Automated Malaria Rapid Diagnostic Test Readers in Health Facilities in the Democratic Republic of Congo: Process, Challenges, and Lessons Learned**
Johanna Karemere, Erick Tshikamba, Erick Mukomena, Curtis Kamwanya, Augustin Guech, Michael Humes, and Yazoumé Yé **10**

**Process and Methodological Considerations for Observational Analyses of Vector Control Interventions in Sub-Saharan Africa Using Routine Malaria Data**
Sarah M. Burnett, Kelly M. Davis, Gudissa Assefa, Christelle Gogue, Levi D. Hinneh, Megan Littrell, Julia Mwesigwa, Okefu O. Okoko, Saraha Rabeherisoa, Musa Sillah-Kanu, William Sheahan, Hannah C. Slater, Perpetua Uhomoibhi, Frederick Yamba, Kelley Ambrose, and Kathryn Stillman **17**

**Bridging the Gap from Molecular Surveillance to Programmatic Decisions for Malaria Control and Elimination**
Monica Golumbeanu, Constant A. V. Edi, Manuel W. Hetzel, Cristian Koepfli, and Christian Nsanzabana **35**

**Optimizing the Delivery of Routine Malaria Data Quality Assessments: A Two-Level Logistic Regression Model to Inform an Institutionalized Approach in Mozambique**
Ann-Sophie Stratil, Maria Rodrigues, Sarmento Armando, Sergio Gomane, Kulssum Mussa, Baltazar Candrinho, and Arantxa Roca-Feltrer **48**

**Improving Coordination to Strengthen Operationalization of Malaria Surveillance and Routine Data Quality: Landscape Analysis of Current Surveillance-Related Initiatives**
Selgün Kayaalpli, Hannah Margaret Edwards, Ann-Sophie Stratil, Julianna Smith, Médoune Ndiop, Balthazar Candrinho, and Arantxa Roca-Feltrer **55**

**Improving the Quality and Use of Malaria Surveillance Data: Results from Evaluating an Integrated Malaria Information Storage System at the Health Facility Level in Selected Districts in Mozambique**
Ann-Sophie Stratil, Neide Canana, Maria Rodrigues, Sarmento Armando, Sergio Gomane, Edson Zandamela, Kevin Baker, Arantxa Roca-Feltrer, and Baltazar Candrinho **59**

**Surveillance for Action: Operationalizing Private Sector Surveillance and Service Delivery across the Malaria Transmission Continuum**
Erica Berlin, Christopher Lourenço, and Stephen Poyer **66**

For more information about
the American Society of Tropical Medicine and Hygiene
please visit us at www.astmh.org

The American Journal of Tropical Medicine and Hygiene is
online at www.ajtmh.org
